# FAMILY CAREGIVERS AT WORK: THE RELATIONSHIP BETWEEN WORK PRODUCTIVITY LOSS AND WORKPLACE SUPPORTIVE MEASURES

**DOI:** 10.1093/geroni/igad104.2835

**Published:** 2023-12-21

**Authors:** Mary Milnamow, Louanne Bakk

**Affiliations:** University at Buffalo - SUNY, Buffalo, New York, United States; University at Buffalo - SUNY, Buffalo, New York, United States

## Abstract

**Background:**

Employment and economic security remain elusive for the majority of working family caregivers, especially for caregivers for older adults with complex health problems. Family paid leave remains unattainable in the United States requiring caregivers to work while providing critical support to older adults making them vulnerable for unemployment and financial hardships. While caregivers experience higher rates of absenteeism and presenteeism at work compared to their non-caregiving counterparts, research pertaining to the impact of supportive measures in the workplace is relatively unknown.

**Methods:**

This exploratory, cross-sectional study examined 1) the relationship between supportive workplace measures (i.e., employment stability, flexible work schedules), and productivity loss, absenteeism and presenteeism; and 2) the relationship between caregiver characteristics (i.e., traditional versus non-traditional) and supportive workplace measures, productivity loss, absenteeism, and presenteeism. Data from 2017 (n=2,652) and 2020 (n=1,938) waves of the National Survey of Caregivers (NSOC) were analyzed using the validated WPAI-CG measure.

**Results:**

Bivariate findings suggest an association between workplace flexibility and productivity loss (p<.001), absenteeism (p<.001), and presenteeism (p<.001); caregivers with flexible hours had greater productivity loss, absenteeism, and presenteeism compared to caregivers without flexible hours. Additionally, caregivers in traditional dyads reported higher work productivity loss (p<.001) and presenteeism (p<.001) compared to non-traditional dyads.

**Conclusions:**

Findings suggest flexible hours may not adequately address workplace issues experienced by family caregivers for older adults. As the number of older adults increases over the next decade, greater understanding of the best way to decrease work challenges for diverse caregivers is critical.

